# Supplementary material for: The Serine Carboxypeptidase-Like Gene SCPL41 Negatively Regulates Membrane Lipid Metabolism in Arabidopsis thaliana
Source: Plants (Basel). 2020 May 29;9(6):696. doi: 10.3390/plants9060696 (PMC7355682; doi:10.3390/plants9060696)
Supplement: Supplementary file 1 [file plants-09-00696-s001.pdf]

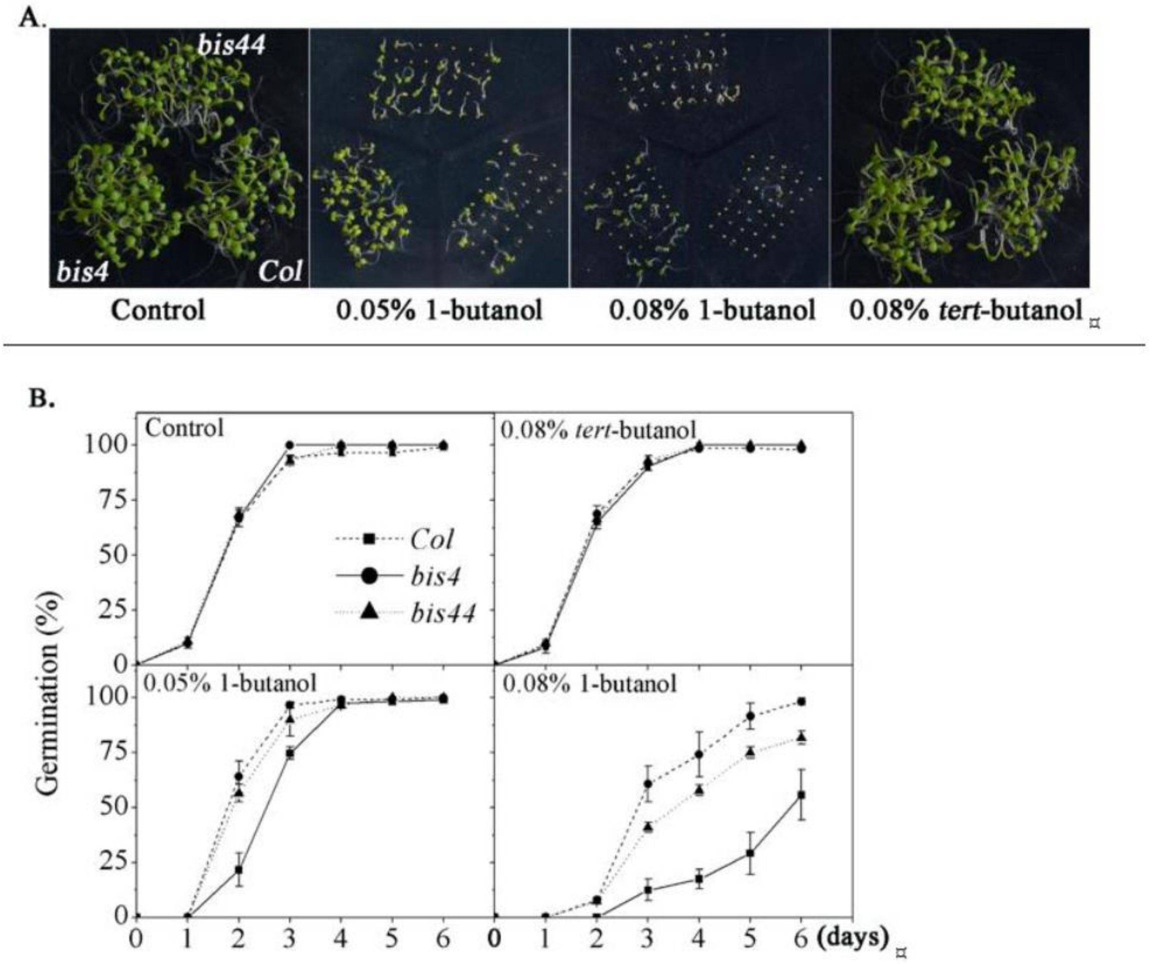

Figure S1. *SCPL41* is required for 1-butanol response. (**A**) Visual comparison of *Col*, *bis4*, *and bis44* seed germination and postgerminative growth after 6 d in the absence or presence of 0.05% 1-butanol, 0.08% 1-butanol, or 0.08% *tert*-butanol. The values are the means ± SDs (*n* = 120). (**B**) Germination rate of *Col* wild type, *bis4* and *bis44* over 6 d in the absence or presence of 0.05% 1-butanol, 0.08% 1-butanol, or 0.08% *tert*-butanol at indicated times.

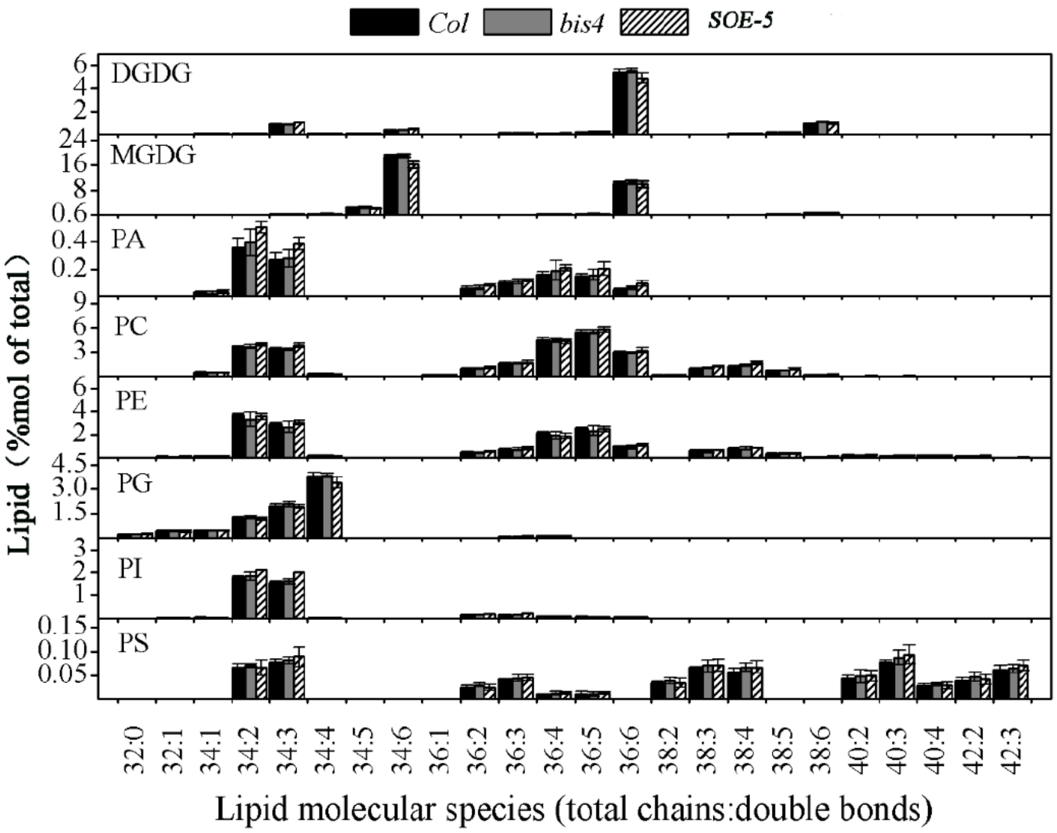

Figure S2. Lipid composition within each head-group class in *Col*, *bis4* and *SOE-5* plants. The values are the means ± SDs (*n* = 4 or 5).
